# Molecular prognostic markers for adult acute myeloid leukemia with normal cytogenetics

**DOI:** 10.1186/1756-8722-2-23

**Published:** 2009-06-02

**Authors:** Tara K Gregory, David Wald, Yichu Chen, Johanna M Vermaat, Yin Xiong, William Tse

**Affiliations:** 1 Division of Medical Oncology, University of Colorado Cancer Center, University of Colorado School of Medicine, Aurora, Colorado, USA; 2 Department of Medicine, Case Western Reserve University, Cleveland, Ohio, USA

## Abstract

Acute myeloid leukemia (AML) is a heterogenous disorder that results from a block in the differentiation of hematopoietic progenitor cells along with uncontrolled proliferation. In approximately 60% of cases, specific recurrent chromosomal aberrations can be identified by modern cytogenetic techniques. This cytogenetic information is the single most important tool to classify patients at their initial diagnosis into three prognostic categories: favorable, intermediate, and poor risk. Currently, favorable risk AML patients are usually treated with contemporary chemotherapy while poor risk AML patients receive allogeneic stem cell transplantation if suitable stem cell donors exist. The largest subgroup of AML patients (~40%) have no identifiable cytogenetic abnormalities and are classified as intermediate risk. The optimal therapeutic strategies for these patients are still largely unclear. Recently, it is becoming increasingly evident that it is possible to identify a subgroup of poorer risk patients among those with normal cytogenic AML (NC-AML). Molecular risk stratification for NC-AML patients may be possible due to mutations of NPM1, FLT3, MLL, and CEBPα as well as alterations in expression levels of BAALC, MN1, ERG, and AF1q. Further prospective studies are needed to confirm if poorer risk NC-AML patients have improved clinical outcomes after more aggressive therapy.

## Introduction

Acute Myeloid Leukemia (AML) is a broad range of disorders that are all characterized by an arrest of maturation along with uncontrollable proliferation of hematopoietic progenitor cells. The French-American-British classification is still widely used in clinical setting that groups AML into 8 subgroups (M0-M7) based on its degree of differentiation and morphology. Due to the heterogenous nature of AML even within specific FAB subtypes, there is a highly variable prognosis among AML patients. The overall 5-year survival rate for AML is still less than 50% in adults and significantly lower in the elderly [1]. The median survival in patients over the age of 65 is less than one year and only 20% of these patients survive two years [2]. Treatment for all subtypes of AML, except the M3 subtype, involves combination chemotherapy and a possible hematopoietic stem cell transplant as part of consolidation therapy. Acute Promyelocytic Leukemia (APL, M3 subtype) is treated with a combination of the differentiation-inducing agent all-trans retinoic acid and chemotherapy resulting in the presumed cure of 75–85% of patients [3]. In general, the prognosis of patients with AML is currently based upon the presence or absence of cytogenetic abnormalities and is divided into favorable, intermediate and unfavorable subgroups (see table 1) [4]. There is heterogeneity within these subgroups, especially the intermediate subgroup, and the age of the patient is also an important prognostic factor. One study estimated the 5 year overall survival (OS) of the favorable subgroup at 55%, the intermediate subgroup at 38% and the unfavorable subgroup at 11% [5]. Patients who have the following cytogenetic abnormalities: inv(16), t(15;17) (the translocation found in APL), or t(8;21) have a favorable prognosis while patients with several other cytogenetic abnormalities including monosomy 5, monosomy 7, 11q23, and complex cytogenetics may have a poor prognosis. However, approximately 40% of AML patients have no identifiable cytogenetic abnormality by using modern cytogenetic and fluorescence in-situ hybridization (FISH) methods. These patients are usually classified as an intermediate risk group. Although the NC-AML patients are currently considered as having an intermediate prognosis, these patients have a wide range of overall survival rates between 24% to 42%. Recently, multiple institutions from Europe and the United States conducted retrospective studies which showed some molecular markers that could identify good and poor risk NC-AML patients and suggest that these patients should be treated accordingly. This review encompasses a discussion of the molecular markers in NC-AML which are mutations (NPM1, FLT3, MLL-PTD, CEPBα) and those that are a function of over-expression (BAALC, MN1, ERG-1, AF1q). These differences in markers of mutation versus over-expression are summarized in Table 1. Furthermore, some of the genetic abnormalities have also been found to be useful for minimum residual disease monitoring and as potential therapeutic targets.

**Table 1:** Genetic Abnormalities in Normal Cytogenetic AML

Name	Prognosis	Prevalence	Expression
NPM-1	Favorable	50–60%	Mutation

FLT3-ITD	Unfavorable	30–40%	Mutation

FLT3-Asp835	Unclear	5–10%	Mutation

BAALC	Unfavorable	65.7%	Over expression

MN1	Unfavorable	50%	Over expression

MLL-PTD	Unfavorable	7.7%	Mutation/over expression

CEBPα	Favorable	15–20%	Mutation

ERG-1	Unfavorable	25%	Over expression

AF1q	Unfavorable	75%	Over expression

### The Nucleophosmin Gene (NPM1)

Mutations of NPM1 have recently been described as one of the most frequent genetic lesions in AML, occurring in 50–60% of adult AML with normal karyotype [6,7]. Additional evaluation has shown that mutations in NPM1 are rare in other risk groups of AML and in one study, no NPM1 mutations were shown in patients with favorable cytogenetics [7]. The NPM1 gene encodes a nucleo-cytoplasmic shuttling protein that regulates the ARF-p53 tumor-supressor pathway [8,9]. Mutations in this gene result in an abnormal accumulation of the NPM1 protein in cytoplasm. Two types of mutations have been described to date. The first and most frequent mutations consists of a 4-nucleotide (nt) insertion (YWTG; YUPAC code) downstream from nucleotide 959; the second is deletion of a GGAGG sequence at positions 965 through 969 and substitution with 9 extra nt (GenBank accession no. NM_002520). Both mutations lead to aberrant cytoplasmic localization of NPM1 as shown after immunostaining with anti-NPM1 monoclonal antibodies. This is caused by open reading frameshift mutations that lead to either the disruption of the NPM1 nucleolar-localization signal or the generation of a leucine-rich nuclear export motif. In all mutated cases, the resulting frameshift led to a product five amino acids longer with the new C-terminal tail CFSQVSLRK, peculiar to the NPM1-mutated product [7].

Recent studies in cell lines and knockout mice have shown that NPM1 is involved in the control of genomic stability and contributes to growth-suppressing pathways through its interaction with ARF. Therefore, the loss of NPM1 expression can contribute to tumorgenesis [10]. Several methods are suitable for detecting NPM1 gene mutation, including molecular and immunohistochemical studies [7,11-13].

NPM1 gene mutations appear to occur more frequently in adult female AML patients [14-16] and also tend to be associated with: a) higher white blood cell count, b) monocytic differentiation (in particular FAB M5b AML subtype) [17], c) wide morphologic spectrum, d) multilineage involvement [9], e) lack of CD34/CD34-negativity [7,9,11], f) normal cytogenetics [18], g) a decreased prevalence of CEBPα mutations [17], h) high frequency of FLT3-ITD gene mutation [18] and i) a trend toward favorable clinical outcome, especially in patients without a FLT3 gene mutation [7,15]. Patients with only an NPM1 mutation exhibit higher complete remission (CR) and significantly better OS [14-16], event free survival (EFS) [15], and disease free survival (DFS) as well as a lower cumulative incidence of relapse [6,16]. The various study outcomes of risk associated with NPM1 status are summarized in Table 2.

**Table 2:** NPM1 Mutant Risk Assessment

Study	Number of NPM1 mutants/total cases studied	Treatment	Demographics of those patients with NMP1 mutations	+ NPM1 mutant assessment of risk
Verhaak, et al [6]	95/275 (34.5%)	Dutch Belgian Hematology Oncology Cooperative Groop (HOVON) protocols	- Median age 47 yo - 60% of those with FLT3 ITD - decreased in those age < 35 yo - 42% of those with WBC >20 K	HR EFS 1.96 DFS 2.0 OS 2.13

Döhner, et al [14]	145/300 (48.3%)	AML Study Group (AMLSG) AML HD93 AML HD98-A	- Increased in M4/M5 - extramedullary LAD - Female predominance - Decreased CD34 antigen expression - Increased LDH - Associated with FLT3 ITD - WBC >20 K - Increased bone marrow blast counts	Odds ratio (OR) after induction CR 2.81

Schnittger, et al [15]	212/401 (52.9%)	German AMLCG99 study	- Associated with FLT3 ITD - Without FLT3, OS and EFS increased - Female predominance	Relative risk (RR) EFS 0.527

Theide, et al [16]	408/1485 (27.5%)	Deutsche Studieninitiative Leukämie (DSIL) AML 96 protocol	- High bone marrow blasts - Female predominance - WBC >20 K - Association with FLT3-ITD mutations	OR OS 0.76 DFS 0.66

Boissel, et al [17]	50/106 (47%)	French Leukemia French Association (ALFA) ALFA90 ALFA9802	- Increased in FAB M4/M5 - 25% with FLT3-ITD - Decreased CEBPA - WBC >20 K	No difference in CR or long term outcomes

Suzuki, et al [18]	64/257 (24.9%)	Japan Adult Leukemia Study Group protocols	- Associated with FLT3-ITD	- NPM1 mutant unfavorable factor for relapse OR 2.106 - NPM1 wild type unfavorable for CR OR 4.908 -NPM1 mutant with FLT3-ITD favorable for CR OR 20.8

Detection of NPM1 gene mutations may be useful in the dissection of the heterogeneous group of AML patients with normal karyotype into prognostically different subgroups [7]. Further, due to their frequency and stability, NPM1 mutations may become a new tool for monitoring minimal residual disease in AML-patients with a normal karyotype [9].

### The Fms-like tyrosine kinase 3 Gene (FLT3)

FLT3 is a tyrosine kinase that is primarily expressed on hematopoietic progenitor cells and functions in the proliferation and differentiation of these cells. FLT3 is the most commonly mutated gene in AML with the mutation occurring in approximately 30–40% of AML patients [19]. The most common mutation consists of an internal tandem duplication (FLT3-ITD) in the juxtamembrane domain of the FLT3 gene. FLT3-ITD results in a constitutively active FLT3 protein that promotes Stat 5 phosphorylation. The net consequence of FLT3/Stat5 constitutive activation is uncontrolled hematopoietic cell proliferation [20]. AML patients who carry the FLT3-ITD mutation appear to have poorer clinical outcomes. Adult patients usually have a higher prevalence of FLT3-ITD than pediatric AML patients. This observation may partially explain why adult AML has a poorer clinical outcome than pediatric AML. Clinically, AML patients with FLT3-ITD tend to have higher WBC counts and an increase percentage of leukemic blasts [21]. A missense mutation in the activation loop (FLT3-ALM) of the second tyrosine kinase domain of FLT3 at Asp835 leads to another common FLT3 mutant (FLT3-TKD) that is found in approximately 5–10% of AML patients [22]. Although the clinical significance of this FLT3 mutation especially in NC-AML is not yet clear, several studies indicate that it is also an adverse prognostic indicator [19,21]. The associated risk of FLT3 status determined by these studies are summarized in Table 3.

**Table 3:** Positive FLT3-ITD Risk Assessment

Study	Number of FLT3 mutants/total cases studied	Treatment	Demographics of those patients with FLT3-ITD	+ FLT3-ITD assessment of risk
Fröhling, et al [21]	119/523 all comers (22.8%) 71/224 NC AML (32%)	AML Study Group (AMLSG) AML HD93 AML HD98-A	- Associated with high WBC - Associated with de novo AML - Increased bone marrow and peripheral blood blasts - Increased LDH	Hazard ratio (HR) Remission duration 2.35

Kainz, et al [23]	26/100 (26%) 16/53 NC AML (30%)	Various protocols	- Increased in M4 (50%) - Increased LDH - WBC >10 K	OR CR 0.31 Relapse rate 8.3 OS 0.17

Ciolli, et al [24]	25/100 (25%)	Various protocols	- WBC > 30 K - Decreased incidence of secondary AML - Female predominance - Increased LDH	HR RFS 3.1 Post remission survival 2.1

Stirewelt, et al [26]	48/151 (31.8%)	Southwest Oncology Group SWOG 9333 SWOG 9500	- High bone marrow blasts - High peripheral blood blasts - WBC >30 K	HR OS 1.35 RFS 1.7

Whitman, et al [27]	23/82 (28%)	CALGB protocol	- All patients evaluated had NC AML, age <60, and de novo AML - Median age 37 yo - N = 8 FLT3^ITD/-^	No clear evidence in difference between groups, but trend towards decreased OS with FLT3^ITD/-^

Thiede, et al [28]	200/979 (20.4%)	Various protocols	- Increased in M5 - WBC >50 K - Increased bone marrow blasts	OR Mut/wt ratio 1 - all ages OS 1.8 DFS 3.2 - age < 60 DFS 4.2 Mut/wt ratio 2 - all ages OS 2.8 DFS 8 - age < 60 DFS6.9

Particularly in AML patients with normal cytogenetics, FLT3-ITD status is important in assessing the prognosis of patients. Several studies have demonstrated that FLT3-ITD in NC-AML patients correlates with an adverse prognosis for both DFS and OS [23-25]. Not only does the presence of FLT3-ITD impart a poor prognosis, but the size of the internal tandem duplication is significant. The duplication can range in size from three to hundreds of nucleotides and longer duplications correlate with a worse OS [26]. In addition to the mutant allele, the status of the wild-type allele in patients with FLT3-ITD has been demonstrated to have prognostic significance. Patients who lack the wild-type allele have a worse prognosis [27]. In those who express the wild-type allele, the ratio of the mutant to wild-type level of FLT3-ITD has a strong correlation to survival. In one study, patients with a high mutant to wild-type ratio (defined as greater than 0.78) had a significantly shorter OS and DFS than those with a lower ratio. The DFS and OS for patients with a lower ratio were no different than the group of patients without FLT3 abnormalities [28].

In addition to mutation of FLT3 and the decreased expression of the wild-type allele, over-expression of FLT3 in the absence of mutation has also been observed in AML patients. Over-expression of FLT3 even in the absence of FLT3-ITD is also an unfavorable prognostic factor for OS [29]. As FLT3-ITD is an adverse prognostic factor, it has been speculated that patients with this genetic abnormality should be considered for more intensive therapy. However, a large study of 1135 adult patients with AML including 25% with FLT3-ITD, there was no improvement in outcome based upon whether or not a patient with FLT3-ITD received a transplant in first complete remission [30].

The association of FLT3 mutations have also been evaluated in patients with favorable cytogenetics (t(8;21), inv16, t(15;17)). FLT3 mutations were noted in 15 of 17 patients studied. Of these patients, 41% of those with t(15;17) were associated with FLT3 mutations and only 9% of cases with inv16. Those with PML-RARα had decreased to no CD11c or HLA-DR expression. However, this study did not illustrate significant correlations with outcomes and FLT3 mutational status [31].

As FLT3 mutations lead to constitutively activated signaling, much work has been performed to develop small molecule FLT3 inhibitors. Unfortunately FLT3 inhibitors have thus far shown disappointing results as remission induction has been short lived. Nevertheless, there is optimism that FLT3 inhibitors may be more efficacious when used in combination therapies [32]. Besides being a useful as a prognostic marker and a therapeutic target, FLT3-ITD has also been used for minimal residual disease monitoring [33].

### The mixed lineage leukemia gene (MLL)

MLL is frequently rearranged in AML and ALL and has been found in combination with greater than 30 different genes. The most frequent rearrangements in the current published series were unbalanced translocations leading to loss of chromosomal material. Overall, loss of 5q and/or 7q chromosomal material seemed the more common event, and losses of 5q, 7q, and 17p in combination were observed in many cases. Overrepresented chromosomal material from 8q, 11q23, 21q, and 22q was found recurrently and in several cases this was due to the amplification of the MLL (located at 11q23) and AML1/RUNX1 (located at 22q22) genes [34]. MLL encodes a histone methyltransferase that plays a role in hematopoiesis by regulating homeobox genes. In mice heterozygous for MLL, both hematopoietic abnormalities are found as well as decreased Hox gene expression [35].

In addition to rearrangements, the MLL gene can also undergo partial tandem duplications of exons 5–11 or exons 5–12 and produce an elongated protein. This abnormal protein, which contains a DNA-binding and transcriptional repression domain, can suppress the expression of the wild-type allele by an unknown mechanism. Interestingly, the silencing of wild-type MLL in blasts positive for MLL-PTD was reversed by DNA methyltransferase and histone deacetylase inhibitors [36]. These findings indicate the potential therapeutic role of the DNA methyltransferase and histone deacetylase inhibitors such as decitabine and valpoic acid in these AML patients.

In one large study of 247 young adult patients with AML, MLL-PTD was found in 7.7% of patients. In this study, MLL-PTD was an adverse prognostic indicator as the median remission duration was 19 months in the absence of MLL-PTD and 7.75 months in its presence [37]. In general, the majority of studies indicate that MLL-PTD is poor prognostic indicator in NC-AML including median survival and relapse-free interval [38]. Additionally, the use of MLL-PTD for minimal residual disease monitoring has been shown to be effective in detecting relapse prior to clinical manifestations [39].

### The CCAT/Enhancer Binding Protein Alpha Gene (CEBPα)

CEBPα is an essential transcription factor for granulocytic differentiation as demonstrated by CEBPα-null mice that lack mature granulocytes [40,41]. Studies have reported N- and C-terminal CEBPα mutations in approximately 15% to 20% of AML [42]. The mutant proteins act in a dominant-negative manner to block DNA binding and transactivation of granulocyte target genes resulting in the failure of granulocytic differentiation [40]. Patients with a CEBPα mutation have higher hemoglobin levels, lower platelet counts, higher blast counts, and are less likely to present with lymphadenopathy or extramedullary leukemia compared to patients without a CEBPα mutation.

Suprisingly, as CEBPα is required for differentiation, mutation of CEBPα is correlated with beneficial effects on remission, CR duration [42], event-free survival [17], DFS [25], and OS [41]. While there is no significant difference in CR rates between patients with and without CEBPα mutations [25,42], mutations are associated with a significantly reduced hazard ratio for death and event free survival [41]. CEBPα mutations appear to be an independent prognostic factor even in the presence of FLT3 and MLL mutations. Studies have shown that there is no significant overlap between the patients with CEBPα mutation and patients with FLT3-ITD or MLL-PTD mutations, suggesting that CEBPα mutations define a distinct biologic subclass of NC-AML [42]. CEBPα mutation is an independent prognostic marker for OS irrespective of age, MLL-PTD, and FLT3-ITD status [43] and is another marker that permits the division of NC-AML into distinct clinical groups [25].

While NPM1, FLT3, MLL-PTD, CEPBα are all mutations noted in NC-AML, additional genetic abnormalities are noted in the form of over-expression. More detailed discussion of BAALC, MN1, ERG-1, and AF1q give insight to over-expression of genes in NC-AML.

### The Brain and Acute Leukemia Cytoplasmic (BAALC)

BAALC has also been found to be an important adverse prognostic factor in NC-AML. Though little is known about the biological function of BAALC, it is highly expressed in hematopoietic precursor cells as well as leukemic blasts and is down-regulated during differentiation. BAALC has been postulated to function in the cytoskeleton network due to its cellular location [44,45]. Several studies have demonstrated that high BAALC expression is a poor prognostic indicator in NC-AML for such factors as OS, DFS, and resistant disease [46,47]. In one study of 86 AML patients with NC-AML, high expression of BAALC was found to be an independent risk factor for both inferior OS (1.7 vs. 5.8 years) and DFS (1.4 vs 7.3 years). Different post-remission strategies among patients with different level of BAALC expression (consolidation, autologous and allogeneic stem cell transplantation) have no influence on OS. However, high BAALC expressive patients who underwent allogeneic stem cell transplantation have lower cumulative relapse rate compared to those who underwent autologous stem cell transplantation [47].

### Meningioma 1 (MN1)

MN1 is an oncoprotein that has been found to function as a transcription coactivator. In AML it has been found as part of the translocation t(12;22)(p13;q11) which leads to the MN1-TEL fusion gene [48]. In animal models, the MN1-TEL fusion gene collaborates with HOXA9 to induce AML [49]. Recently, high levels of expression of MN1 have been found to be a prognostic marker in NC-AML. Though the exact function of MN1 in hematopoietic cells is unclear, it is another protein that is highly expressed in hematopoietic cells and is down-regulated during differentiation. In a study of 142 adult patients with NC-AML, high MN1 expression was significantly related to unmutated NPM1, poor response to initial induction chemotherapy, high relapse rate, risk free survival, and OS. In multivariate analysis, high MN1 expression was an independent prognostic marker [50].

### The ETS-related gene (ERG)

ERG is a member of the ETS family of transcription factors. High ERG expression is associated with the upregulation of many genes which are involved in cell proliferation, differentiation, and apoptosis [51]. The ERG gene is a recently identified molecular marker predicting adverse outcome of NC-AML patients. Over-expression of the ERG gene was first discovered in patients with complex karyotypes and abnormal chromosome 21 [34,52]. Marcucci, et al showed that in patients less than 60 years old with de novo NC-AML, those patients expressing the highest levels of ERG (the top 25%) have a worse cumulative incidence of relapse (CIR) and OS. In this analysis, ERG over-expression predicted a shorter survival only in patients with low BAALC expression. Though more study is needed to confirm these results, ERG over-expression in NC-AML not only predicts an adverse clinical outcome, but also appears to be associated with a specific molecular signature [51].

As the ERG gene is located on chromosome 21, it has been speculated that ERG expression may play a role in the pathogenesis of acute leukemia in patients with Down's syndrome. Patients with Down's syndrome are known to have a higher incidence of acute leukemia [53]. The high ERG expression may also be related to acute megakaryoblastic leukemia (FAB-M7) which is associated with trisomy 21 [54].

### AF1q expression

Two small studies conducted by Tse et al suggest that elevated AF1q expression is associated with poor outcomes both in pediatric AML and adult myelodysplastic syndrome (MDS) [55,56]. In the pediatric study, AF1q expression in AML patients varied from 0 to 154-fold compared with normal marrow and increasing AF1q expression level was associated with worsening survival with a hazard ratio of 1.02 per fold in AF1q expression (p = 0.032). High AF1q expression was related to poor survival in univariate and multivariate models without association with any specific adverse cytogenetics [55]. The AF1q expression levels in the MDS study (total of 47 patients) suggested a statistically significant correlation with IPSS and AF1q expression level in high risk MDS [56]. Consistent with the findings in the pediatric AML study, MDS patients with high AF1q expression have an increased hazard ratio of death from MDS and relapse after allogeneic stem cell transplantation with correlation of specific poor cytogenetics. These observations led to a hypothesis that elevated AF1q expression might serve an adverse molecular marker for poor prognosis in AML patients with normal cytogenetics.

Recently, Strunk et al examined AF1q expression in 290 adult NC-AML patients (aged < 60). They found NC-AML patients with low AF1q expression (AF1q^low^) had better OS (p = 0.026) and CR rate with initial induction chemotherapy (p = 0.06) compared to high AF1q expressing patients (AF1q^high^). The AF1q^high ^patients had a significantly greater incidence of concurrent FLT3-ITD. A subgroup of the AF1q^high ^patients who received allogeneic stem cell transplantation (SCT) had a significant better relapse-free survival compared to patients who received chemotherapy/autologous SCT (p = 0.04). This suggests that high AF1q expression is a poor prognostic marker for adult NC-AML patients and may help direct post-induction treatment strategies.

### Gene expression Profiling

Normal cytogenetics are detected pretreatment in approximately 45% of patients with de novo acute myeloid leukemia; thus, this constitutes the single largest cytogenetic group of AML. Recently, molecular genetic alterations with prognostic significance have been reported in these patients. They include internal tandem duplication of the FLT3 gene, partial tandem duplication of the MLL gene, mutations of the CEBPα and NPM1 genes and aberrant expression of the BAALC, ERG and MN1 genes. Additionally, gene-expression profiling has been applied to identify prognostically relevant subgroups [57].

Gene expression studies have identified that the majority of NC-AML patients fall into specific clusters that exhibit similar gene expression profiles. The identification of these clusters may not only be useful for diagnostic purposes, but also may help guide prognosis and therapeutic approaches. For example, NC-AML patients were found to up-regulate class I homeobox A and B gene families [58]. In another study, DNA microarray experiments identified two distinct subgroups of NC-AML including one that was closely related to the gene signatures observed in AML with translocations. In this study, NC-AML patients in the "translocation-like" group had a superior prognosis to the other group [57]. Similarly, a separate study also found two distinct gene expression clusters in NC-AML patients with significantly different survival. Using a panel of 133 genes, it was possible to predict the clinical outcome of NC-AML patients [59]. NC-AML patients in the cluster with worse survival were more likely to harbor FLT3 mutations and were more commonly diagnosed with specific AML subtypes (FAB M1 and M2). Bullinger's observation was recently validated by a Cancer and Leukemia Group B (CALGB) study that used a different microarray platform and had a longer follow up [60]. Gene-expression profiling allows a comprehensive classification of AML that includes previously identified genetically defined subgroups and a novel cluster with an adverse prognosis [59]. In the future, gene expression profiling studies will likely play a role clinically in molecularly risk stratifying NC-AML patients as well as further elucidating the biology of NC-AML.

### Minimal Residual Disease

Minimal residual disease (MRD) can provide an early indication of potential relapse in AML post-treatment. There has been some initial evaluation of the utility of FLT3-ITD, CEBPα, ERG, NPM1, and MLL in MRD whereas AF1q, MN-1, and BAALC have not. The impact of FLT3 was analyzed in 11 patients. All of the six patients with positive quantitative real-time polymerase chain reaction (RQ-PCR) post-treatment eventually relapsed [33]. Real-time quantitative PCR has also been used to evaluate MRD in patients carrying NPM1 mutations at time of diagnosis. Decreasing NPM1 copy number correlated with response to therapy and in four cases followed post-therapy, rising copy number preceded hematological relapse [13]. Additional evaluation in the post-transplant setting showed that all patients who remained NPM1 mutant positive after transplant relapsed and all those who had increases in mutation copies post-transplant relapsed as well [61]. Comparison of CEBPα mutational status between diagnosis and relapse in AML was first investigated by Tiesmeier et al [62]. Two of 26 patients that relapsed had mutated CEBPα which persistent post-treatment suggesting a concordance between presentation and relapse. As approximately 60% of CEBPα mutations are insertion or deletions, they are an amenable to MRD monitoring by RQ-PCR [63]. One case study have also reported the utility of RT-PCR for detecting ERG MRD. The patient had a negative ERG fusion gene after transplant which then recurred at time of relapse [64]. A larger analysis of 19 patients conducted by Kong et al. noted four types of TLS/FUS-ERG chimeric transcripts via RT-PCR. The transcripts were detectable at diagnosis as well as during remission and relapse suggesting resistance to conventional chemotherapy [65]. Finally, RT-PCR has also been used to evaluate expression levels of partial tandem duplications in the MLL gene. Expression levels in 16 patients were analyzed at time of diagnosis and at relapse and found to be equivalent. Additionally, molecular relapse was detected 35 days before clinical relapse in two patients [39]. Thus, MLL-PTD was suggested as a target for MRD detection. Currently, MRD monitoring via these molecular markers is not commercially available to the community physician, but these studies provide insight to their future potential in MRD monitoring.

## Conclusion

Though NC-AML comprises the single largest subgroup of AML, these patients pose considerable challenges in diagnosis, risk stratification, and post-treatment monitoring for minimal residual disease. Gene expression and profiling studies have shown that NC-AML is very heterogeneous at the molecular level. Multiple studies have shown that NC-AML patients usually exhibit two or more genetic aberrations. While assessment of these molecular prognostic markers is not widely available to the community physician outside of a clinical trial, future studies will help to further validate the prognostic importance of these altered genetic abnormalities in systemic multivariate analysis in the NC-AML patients. Once the prognostic importance of these genetic abnormalities is clear, it may be possible to appropriately tailor the aggressiveness of therapy to NC-AML patients. Further, these studies also have the potential to identify novel therapeutic targets that may be used to design targeted therapies. In the future, specific genetic abnormalities may be profiled in AML patients in a similar manner to the immunophenotyping that is currently done by flow cytometry to obtain information on accurate diagnosis, prognosis, and disease monitoring.

## Competing interests

The authors declare that they have no competing interests.

## Authors' contributions

All authors participated in the drafting of the manuscript. TG edited the manuscript and WT and TG read and approved the final manuscript.
